# Expression of MicroRNA-29a Regulated by Yes-Associated Protein Modulates the Neurite Outgrowth in N2a Cells

**DOI:** 10.1155/2017/5251236

**Published:** 2017-09-12

**Authors:** Chunye Tan, Changlin Yu, Zhiwen Song, Hongjun Zou, Xu Xu, Jinbo Liu

**Affiliations:** Spine Surgery, The Third Affiliated Hospital of Soochow University, No. 185 Juqian Street, Changzhou, Jiangsu 213003, China

## Abstract

Yes-associated protein (YAP) is proved to increase miR-29a in the present study, but the relevant molecular mechanism is not clear. Also, growing evidence indicates that the high-level miR-29a promotes the neurite outgrowth by decreasing PTEN (phosphatase and tensin homologue deleted on chromosome 10). Results show that the expression of miR-29a increases but the PTEN decreases during transfecting the N2a cells with the YAP plasmid. Meanwhile, the advancement of neurite outgrowth is presented via using multiple methods to detect the expression of GAP-43 and NF-200, which have a strong association with neurite outgrowth. The expression of miR-29a, GAP-43, and NF-200 shows an opposite tendency compared to the PTEN when YAP is downregulated. By treating N2a cells with miR-29a mimic and inhibitor, we also find the same conclusion. For in silico analysis of miR-29a, its promoter may have a binding site for YAP. Based on a luciferase reporter assay and a chromatin immunoprecipitation (ChIP) experiment, we demonstrate that YAP could increase the expression of miR-29a by targeting the promoter of miR-29a. In conclusion, the results identify that YAP promotes the neurite outgrowth via targeting the promoter of miR-29a, and it may be an effective therapeutic medicine for the neural disease.

## 1. Introduction

Growing studies reveal that the Hippo signaling pathway plays a crucial role in regulating tissue and organ development [1, 2]. YAP is a major downstream effector of the mammalian Hippo pathway, which is phosphorylated and inhibited by the Hippo pathway component Lats [3, 4]. Previous studies about YAP mainly focus on the function such as proliferation, apoptosis, and angiogenesis [5]. But its function in the nervous system is not clearly clarified. However, we acquire significant information that YAP increases the expression of miR-29 family, which links YAP and neural growth. More and more information indicates that miRNAs are the important mediators of axonal regeneration and neurodevelopment [6], such as miR-17 [7] and miR-29 [8, 9]. Notably, miR-29s have a close relationship with axonal growth, including miR-29a, miR-29b, and miR-29c [10]. Among them, miR-29a as a newfound regulatory factor is confirmed to target PTEN on promoting neurite outgrowth. PTEN which is a target of miR-29 family is closely combined with neurite outgrowth [11–13]. Growing evidences support that PTEN inhibition facilitates neuronal development [14–16]. Further study shows that miR-29a promotes neurite outgrowth by downregulating PTEN expression and increasing the Akt phosphorylation level. Our data further confirm that YAP could inhibit PTEN expression by overexpressing miR-29a. TEAD as an important transcription factor plays a crucial role in the regulation of gene expression [17]. Also, TEAD which emerged as the main partner of YAP on DNA has also been reported to interact with YAP to regulate gene expression [18, 19].

In our research, we confirm that YAP facilitates neurite outgrowth in N2a cells. Also, results show that miR-29a expression is increased and PTEN is decreased during N2a cells treated with YAP. Among them, we demonstrate that YAP decreases the PTEN expression via overexpressing the miR-29a. Our experiments further confirm that YAP binds the promoter of miR-29a to increase its expression. Furthermore, we indicate that the binding sites are TEAD regions. In short, YAP increases the expression of miR-29a and promotes neurite outgrowth by modulating the PTEN expression and the Akt phosphorylation.

## 2. Materials and Methods

### 2.1. Cell Culture

Mouse neuroblastoma (N2a) cells (obtained from the Type Culture Collection of the Chinese Academy of Sciences, Shanghai, China) are grown in Dulbecco's minimum essential medium (DMEM)/F12 (Hyclone, Logan, UT, USA) medium with 10% fetal bovine serum (FBS, Gibco). All cells are cultured in an incubation room gassed with 5% CO2 at 37°C. Cells are seeded into six-well plates at a density of 5 × 10^4^ cells/well.

### 2.2. Real-Time PCR

Total RNA from N2a cells is isolated by using Trizol reagent. Then it is converted to cDNA via using a reverse transcriptase kit (TAKARA) according to the manufacturer's manual. The resultant cDNA is diluted at 1 : 5 for subsequent research. The real-time PCR experiment is worked according to the manufacturer's protocol and the real-time PCR SYBG Green q-PCR super-mix (Bio-Rad, USA) for rat GAPDH, PTEN, NF-200, and GAP-43 (from Sangon Biotech, China) is used. The sequences of primers are displayed in Table 1.

### 2.3. Western Blot Analysis

The cultured cells are washed 3 times with cold Phosphate Balanced Solution (PBS). Then the Radio-Immunoprecipitation Assay (RIPA) lysis buffer is added to the cells at 4°C for 10 minutes. The mixture is centrifuged at 12,000 rpm, 4°C for 10 minutes. 10% Sodium Dodecyl Sulphate-Polyacrylamide Gel Electrophoresis (SDS-PAGE) is used to separate the equal amounts of the protein. When the proteins are transferred to the Polyvinylidene Fluoride (PVDF) membrane, the 5% BSA is used to bind the protein of the membrane for 1 h at room temperature. Subsequently, the membrane is incubated with primary antibodies which are the PTEN, NF-200, and GAP-43 (Cell Signal Technology, BSN, USA) at 4°C overnight. After washing 3 times with the PBS, a goat-anti-rabbit IgG-HRR secondary antibody (Thermo Pierce, MA, USA) is used for incubating the membrane at room temperature for 2 h. Protein levels are normalized to GAPDH by using a mouse monoclonal anti-GAPDH antibody (Thermo Pierce, MA, USA).

### 2.4. Cell Transfection

Gain-of-function experiments of miR-29a are performed with the miR-29a mimics, which are the pre-miRNA precursor molecules. The anti-miRNA inhibitors (mature sequence: UAACCGAUUUCAGAUGGUGCUA) are used for loss-of-function analysis. The* Caenorhabditis elegans* miR-67 served as a negative control (cel-miR-67 mimic mature sequence: UCACAACCUCCUAGAAAGAGUAGA; cel-miR-67 inhibitor mature sequence: UCUACUCUUUCUAGGAGGUUGUGA). 100 nM miRNA mimics and inhibitors are mixed with Lipofectamine 2000 according to the manufacturer's instructions.

### 2.5. Plasmid Construction and shRNA Preparation

The shRNA against mouse YAP is obtained from the RNAi Consortium. The sequence is listed in the 5′ to 3′ direction as follows: sh-YAP-A: CCGGACTTGGAGGCGCTCTTCAATGCTCGAGCATTGAAGAGCGCCTCCAAGTTTTTTG; sh-YAP-B: AATTCAAAAAACTTGGAGGCGCTCTTCAATGCTCGAGCATTGAAGAGCGCCTCCAAGT. The control shRNA is designed to target a gene which does not express endogenously protein called green fluorescent protein (GFP). The plasmid containing the promoter of miR-29a or the negative control vector, which contain the green fluorescence protein sequence, is purchased from the Jikai (Shanghai Genechem Co., Ltd.).

### 2.6. Target Prediction

Three online programs, NCBI (http://www.ncbi.nlm.nih.gov/), UCSC (http://www.genome.ucsc.edu/), and Ensembl (http://www.ensembl.org/index.html), are used for looking for the promoter of mus-miR-29a. Also, the JASPAR (http://jaspar.genereg.net/) and TFSEARCH (https://www.cbrc.jp/research/db/TFSEARCH.html) are applied to predict the transcription factor binding site.

### 2.7. Immunocytochemistry

Morphological analysis of cells after being treated with YAP is observed under a fluorescent microscope. After 72 h with the YAP transfection, we wash the cells 3 times with PBS. Subsequently, 4% paraformaldehyde is used for 2 h at room temperature. After washing 3 times with PBS, 0.3% Triton X-100 in PBS is added to the cells for 10 minutes at 37°C. After washing with the PBS, we use the primary antibodies to incubate the cells at 37°C for 3 h. The primary antibodies are rabbit anti-GAP-43 (1 : 300, NO: BA0878, BOSTER, China) and mouse anti-NF-200 (1 : 300, NO: ab77745, Abcam, UK). Then, we wash the cells for 3 times with PBS and the cells are incubated with an appropriate secondary antibody for 1 h at 37°C. The GAP-43 antibody group is added to the Cy3-labeled goat-anti-mouse IgG antibody and the NF-200 antibody group is used with the Cy3-labeled goat-anti-rabbit IgG antibody (1 : 100, BA1032, Boster, China). Cell nuclei are performed with DAPI at 37°C for 10 minutes. At last, we observe the cells by using an inverted fluorescent microscope.

### 2.8. Luciferase Reporter Assay

The cell suspension of proper concentration is made by trypsinized cells in logarithmic growth period; then cells are seeded at 2 × 10^4^ cells per well in a 24-well plate and incubated in a humidified 37°C incubator with 5% CO2 till 60% confluence. Using X-tremeGENE HP (ROCHE) for transfection procedures, (a) for every 1 ug plasmid, 2 ul X-tremeGENE HP reagent is required; X-tremeGENE HP and plasmids at this ratio in 100 ul opti-MEM are mixed thoroughly, and the compound is incubated at room temperature for 20 minutes; (b) the medium in the plate is replaced with 200 ul opti-MEM; (c) the mixture of X-tremeGENE HP reagent and plasmids is added to the well, after that it is incubated in a humidified 37°C incubator with 5% CO2 for 5-6 hours, and then we replace the mixture with complete medium containing 10% FBS; (d) after transfection for 24–48 h, we observe the expression of fluorescent protein marker by the plasmids to determine transfection efficiency. At last, the luciferase activities are measured with the dual-luciferase reporter system (E1910, Promega) according to the manufacturer's instructions.

### 2.9. ChIP

After N2a cells are treated with YAP, we perform a ChIP assay with the Chromatin Immunoprecipitation Assay Kit (Temecula, CA 92590) the next day. N2a cells are cross-linked by 1% formaldehyde (Solarbio, China) soon after unreacted formaldehyde is quenched by 2.5 M glycine (Nanjing SunShine Biotechnology, China). The sonication, using 25% power, working 2.5 seconds, and pausing 8 seconds, 3 minutes totally, is used to shear the chromatin DNA which mainly contains 200–500 base pairs on ice (TZL-150wk, Suzhou Percival, China). Each tube contained 900 ul dilution buffer containing Protease Inhibitor Cocktail II (Shanghai Yuanye Biotechnology, China) which is mixed with 100 ul chromatin DNA. Then we add anti-RNA polymerase II (ab817, Abcam, UK), normal mouse IgG (*β*-Actin, #3700, Cell Signaling, USA), and YAP (#14074, Cell Signaling, USA) antibody into the tubes, respectively. The samples are incubated overnight at 4°C with rotation and then 60 ul Protein A Agarose is added to each tube and the compound is incubated for 2 h at 4°C with rotation. The Protein A Agarose is pelleted by brief centrifugation at 700 rpm for 1 minute and the supernatant fraction is removed. We wash the Protein A Agarose/antibody/chromatin complex by resuspending the beads with 1 ml cold buffers in the order listed. And the complex is incubated for 10 minutes on a rotating platform; subsequently, it is briefly centrifuged at 700 rpm for 1 minute. Then we carefully remove the supernatant fraction. For each tube, 200 ul elution buffer is prepared. For input tube, 200 ul elution buffer is added and set aside at room temperature. 100 ul elution buffer is added to each tube containing the antibody/agarose complex and mixed by flicking tube gently; then it is incubated at room temperature. The agarose is pelleted by brief centrifugation at 700 rpm for 1 minute and the supernatant is collected into new microcentrifuge tube, the same operation is repeated, and the elution is combined. To all tubes, 8 ul 5 M NaCl is added and incubated at 65°C overnight to reverse the DNA/protein cross-links. Then to all tubes, 1 ml RNase A is added and incubated for 30 minutes at 37°C and 4 ul 0.5 M EDTA, 8 ul 1 M Tris-HCl, and 1 ul proteinase K are added to each tube to incubate at 45°C for 1 hour. At last, the Sangon Biotech DNA kit (B518221, Sangon Biotech) is used to purify the DNA and the purified product is performed for real-time PCR. The purified DNA is eluted and stored at −20°C.

### 2.10. Statistical Analysis

The data obtained from three independent experiments are presented. Significance of the data is assessed via one-way ANOVA, and *P* < 0.05 is considered statistically significant. We use a Student's *t*-test to detect the significant differences between the treated group and the control group.

## 3. Results

### 3.1. miR-29a Is Upregulated and PTEN Is Downregulated after Being Treated with YAP

**Figure 1:**
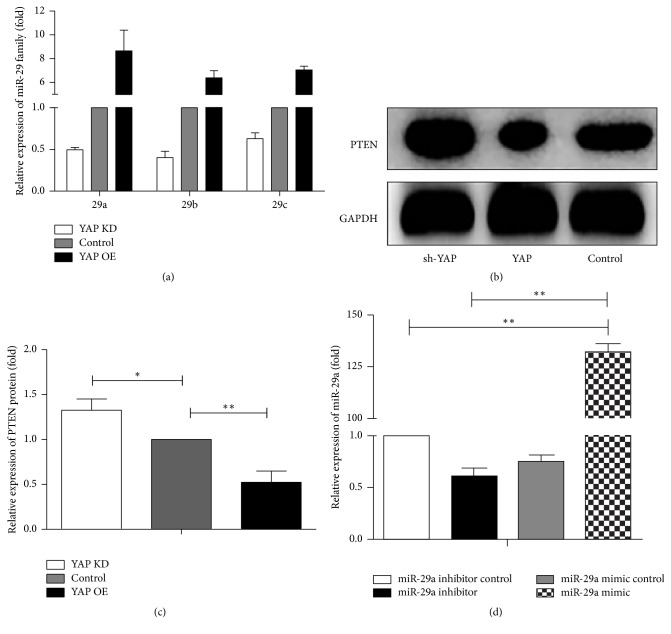
YAP increases the expression of miR-29a and decreases PTEN in the N2a cells. (a) Real-time PCR analysis shows relative expression of miR-29 family with YAP overexpression and knockdown in N2a cells. The control group is transfected with a blank vector and the KD group is treated with sh-YAP. Mean ± SD (*n* = 3). (b) There are changes in levels of PTEN protein expression when YAP OE and YAP KD, and the GAPDH served as an internal control. The blank vector is used as a control. (c) There is the quantification of densitometric levels of PTEN. Mean ± SD (*n* = 3). An ANOVA test is used here. ^*∗∗*^*P* < 0.01, ^*∗*^*P* < 0.05. (d) Real-time PCR test for the relative expression of miR-29a mimic and miR-29a inhibitor. We use miR-29a inhibitor control as the baseline. Mean ± SD (*n* = 3). An ANOVA test is used here. ^*∗∗*^*P* < 0.01, ^*∗*^*P* < 0.05.

It is demonstrated that YAP plays an important role in promoting organ growth, cell proliferation, expanding neural progenitors, and so on [20]. Growing evidences show that YAP overexpression can modulate the miR-29 family and the miR-29 family suppresses the expression of PTEN [21]. Then we use a real-time PCR to analyze it 2 days after YAP overexpression and knockdown (Figure 1(a)). Among them, the miR-29a level has a most prominent change. So the miR-29a is chosen for further study. To test whether the protein of PTEN is altered in N2a cells at the same time, we perform a western blot experiment to examine the PTEN expression (Figures 1(b) and 1(c)). As shown in the result, the protein of PTEN decreases when overexpressing YAP. Conversely, YAP knockdown results in the increasing level of the PTEN. Indeed, we observe an increase in the level of miR-29a and a decrease of PTEN when YAP overexpression.

### 3.2. YAP Regulates the Expression of PTEN via miR-29a

**Figure 2:**
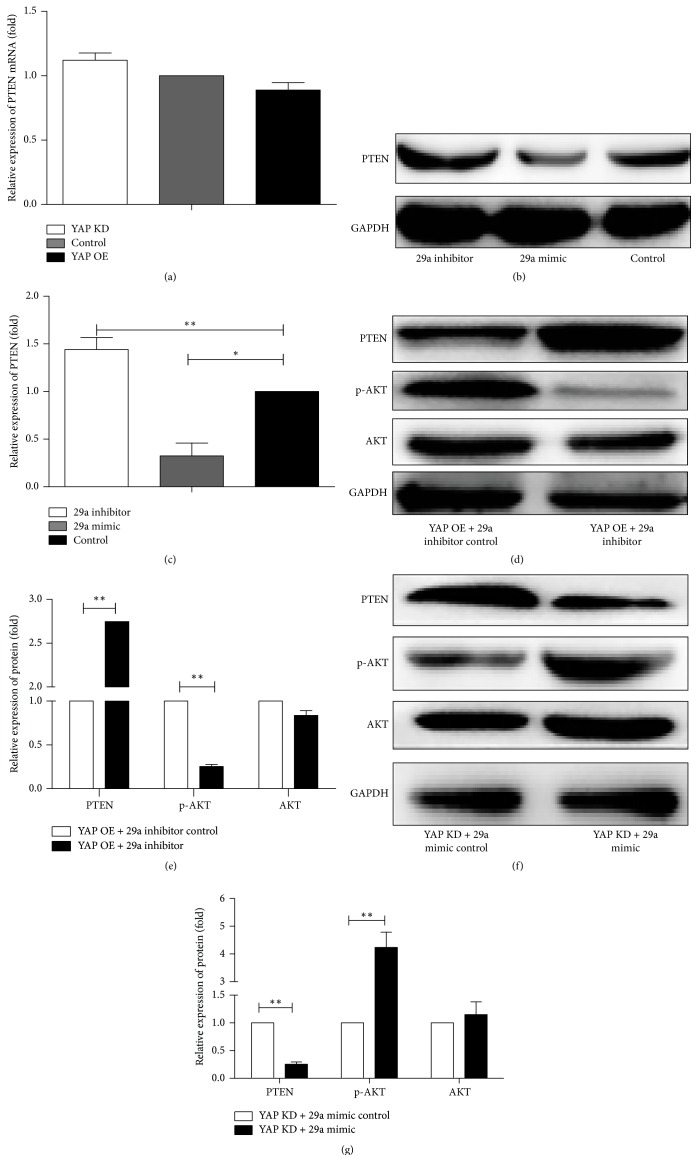
YAP decreases PTEN expression via miR-29a. (a) The PTEN expression at the mRNA level is similar between the three groups. Mean ± SD (*n* = 3). (b) Western blot analysis of PTEN expression shows that miR-29a OE group can decrease the PTEN and miR-29a KD group can increase it. (c) There is the quantification of densitometric levels of PTEN. Mean ± SD (*n* = 3). An ANOVA test is used here. ^*∗∗*^*P* < 0.01, ^*∗*^*P* < 0.05. (d, f) miR-29a mediates the expression of the PTEN protein. YAP overexpression and miR-29a inhibitor have increased protein levels of PTEN and decreased p-Akt. YAP knockdown and miR-29a mimic have decreased PTEN expression and increased p-Akt. There is no change of Akt protein. (e, g) There are the quantification of densitometric levels of PTEN, Akt, and p-Akt. Mean ± SD (*n* = 3). Student's *t*-test is used here. ^*∗∗*^*P* < 0.01.

To investigate whether YAP targets PTEN directly, we use a real-time PCR to test the mRNA of PTEN. As shown in Figure 2(a), the PTEN expression at the mRNA does not have a significant change, just the opposite; the PTEN at protein level in N2a cells is decreased after the transfection of YAP. Thinking of the mechanism where the miRNAs reduce the protein level and increase the level of miR-29a, we make a bold hypothesis that YAP regulates the PTEN via miR-29a. To confirm the hypothesis, we transfect the miR-29a mimic and the miR-29a inhibitor into N2a cells to test the expression of PTEN (Figures 2(b) and 2(c)). The results show the negative correlation of the miR-29a and the PTEN expression. It is similar to N2a cells transfected with YAP and results remind us with the possible relationship between the YAP and the PTEN. Then we transiently transfect the miR-29a mimic in YAP knockdown (YAP KD) cells and the miR-29a inhibitor in YAP overexpression (YAP OE) cells. A protein analysis indicated that the expressions of PTEN, p-Akt, and Akt are changed (Figures 2(d), 2(e), 2(f), and 2(g)). We observe a significant increase of PTEN and a decrease of p-Akt when the miR-29a is blocked by the miR-29a inhibitor in YAP OE cell (Figures 2(d) and 2(e)). Consistently, improvement of the miR-29a by the miR-29a mimic causes a decrease of PTEN and an increase of p-Akt in YAP KD cells (Figures 2(f) and 2(g)). At the same time, Akt does not have a change in two experiments. These data support that miR-29a plays a leading role in regulation of PTEN.

### 3.3. YAP Facilitates Neurite Outgrowth in N2a Cells

**Figure 3:**
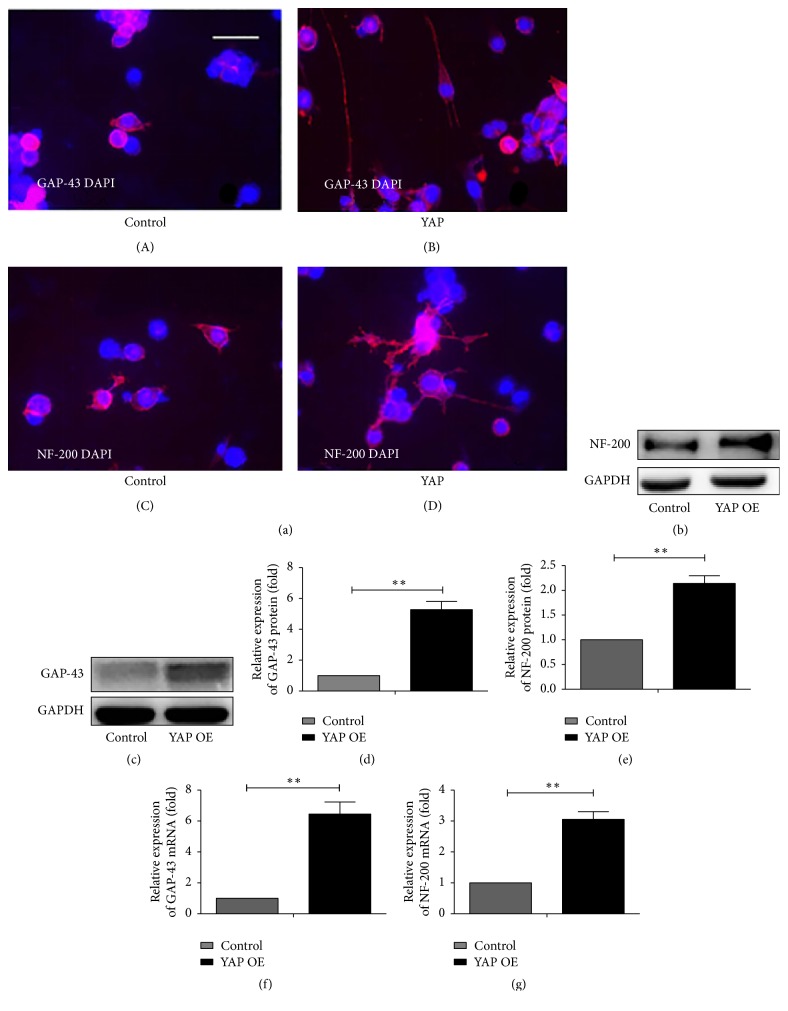
YAP promotes the neurite outgrowth of N2a cells. (a) GAP-43 and NF-200 expression are measured in N2a cells. Representative immunofluorescence is observed with anti-GAP-43 and anti-NF-200 treated with YAP (B, D) and control (A, C). The blank vector is used as a control. Mean ± SD (*n* = 3). Scale bar = 50 *μ*m. (b, c) Western blot analysis shows the protein expression of GAP-43 and NF-200 for treatment group and control group. There are obvious differences in neural protein expression. (d, e) There is the quantification of densitometric levels of GAP-43 and NF-200. Mean ± SD (*n* = 3). Student's *t*-test is used here. ^*∗∗*^*P* < 0.01. (f, g) Real-time PCR indicates that YAP group expresses NF-200 and GAP-43 higher than control group. Mean ± SD (*n* = 3). Student's *t*-test is used here ^*∗∗*^*P* < 0.01.

In view of the fact it is correlated with the axon development that YAP improves the miR-29a expression and decreases the PTEN expression, we further research the role of YAP in neurite outgrowth. In previous studies, GAP-43 and NF-200 have a close connection with neurite outgrowth [22, 23]. So we detect the expression of the two proteins after being transfected with YAP. We perform two groups of cells: one is transfected with YAP and the other is the control group. Immunofluorescence technique is used to examine the expression of GAP-43 and NF-200. Then we find a significant increase in YAP group compared to the control group (Figure 3(a)). To test the validity of this result, we detect the expression of two proteins by western blot analysis. As the results show, both of two proteins are increased after being treated with YAP (Figures 3(b), 3(c), 3(d), and 3(e)). Consistently, real-time PCR analysis also shows the significant increase at mRNA level of them (Figures 3(f) and 3(g)). From the morphology, gene, and protein, we find that YAP does promote the neurite outgrowth in N2a cells.

### 3.4. YAP Is Connected with PI3K/Akt Signaling Pathway

**Figure 4:**
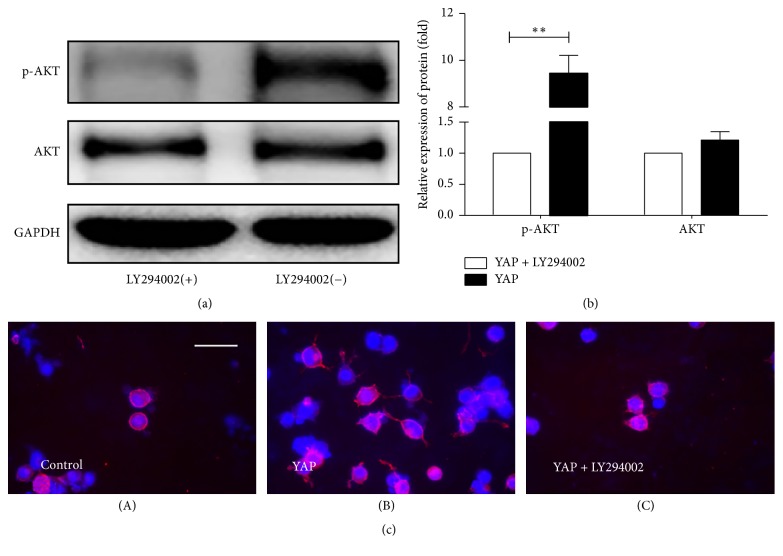
The effect of YAP and Akt pathway on axonal growth. (a) There is the expression of Akt and Akt phosphorylation in YAP OE cell treated with or without PI3-kinase inhibitor (LY294002). (b) There is the quantification of densitometric levels. Mean ± SD (*n* = 3). The figure shows the similar expression of Akt protein in LY294002 (+) group and LY294002 (−) group, but p-Akt almost disappeared in LY294002 (+) group. Student's *t*-test is used here. ^*∗∗*^*P* < 0.01. (c) Immunofluorescence is used to observe the neurite outgrowth at different conditions. Original cells are showed with no axon outgrowth on the first day (A). After 72 hours of treatment with YAP only, we see some growth in axon outgrowth (B). But treated with YAP, while adding LY294002 at the same time, we find a significant inhibitory effect in axon outgrowth compared with the YAP group (C).

**Figure 5:**
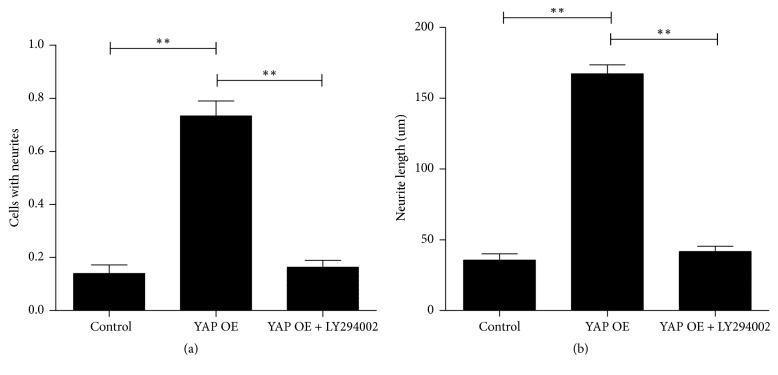
Analysis of neurite outgrowth of N2a cells. Immunofluorescence is used to observe neurite outgrowth of N2a cells. (a) There is a significant difference between the YAP overexpression group and the YAP overexpression group + LY294002 group, also between the YAP overexpression group and the control group. The group of treated with YAP has an obvious increase of the axon; however, the neuritis is shortened obviously during adding LY294002. The blank vector is used as a control. Mean ± SD (*n* = 3). An ANOVA test is used here. ^*∗∗*^*P* < 0.01. (b) In addition, the maximum neurite length is identified as the neurite length per cell. It shows the huge difference that YAP overexpression group is much longer than the YAP overexpression +LY294002 group and the control group. Mean ± SEM (*n* = 3). An ANOVA test is used here. ^*∗∗*^*P* < 0.01.

It has been reported that miR-29a promotes the growth of axons associated with PI3K/Akt signaling pathway [24]. Also we have already known some information, the first is that the YAP increases the expression of the miR-29a, and the second is that the miR-29a facilitates neurite outgrowth. In view of these results, we assume that YAP promotes the neurite outgrowth via PI3K/Akt signaling pathway. Then we perform the N2a cells with or without LY294002, a PI3K inhibitor. Through the protein analysis, we find that the inhibitor does not affect the protein of Akt but blocks the phosphorylation of Akt completely (Figures 4(a) and 4(b)). On the other hand, we research the effect of LY294002 on the growth of axons. We divide the N2a cells treated with YAP into two groups. One group adds the LY294002 to block PI3K/Akt signaling pathway. Another group without LY294002 is used for comparison. Immunofluorescence technique is used to observe the growth of axons. During blockage of the PI3K/Akt signaling pathway, there is almost no growth of the axons compared to the original cells (Figure 4(c)(C)). However, the group without LY294002 has an obvious growth of axons compared to the group treated with LY294002 (Figure 4(c)(B)). And we perform an analysis of neurite outgrowth of the N2a cells. There are more than 100 cells which are selected in at least 10 randomly fields under a fluorescent microscope. It is defined as positive for neurite outgrowth if the neurites of cells are greater than the length of their bodies. The maximum neurite length is identified as the neurite length per cell. It is measured by Image J and is recorded. The outcome is showed in Figures 5(a) and 5(b).

### 3.5. YAP Binds the Promoter of the miR-29a

**Figure 6:**
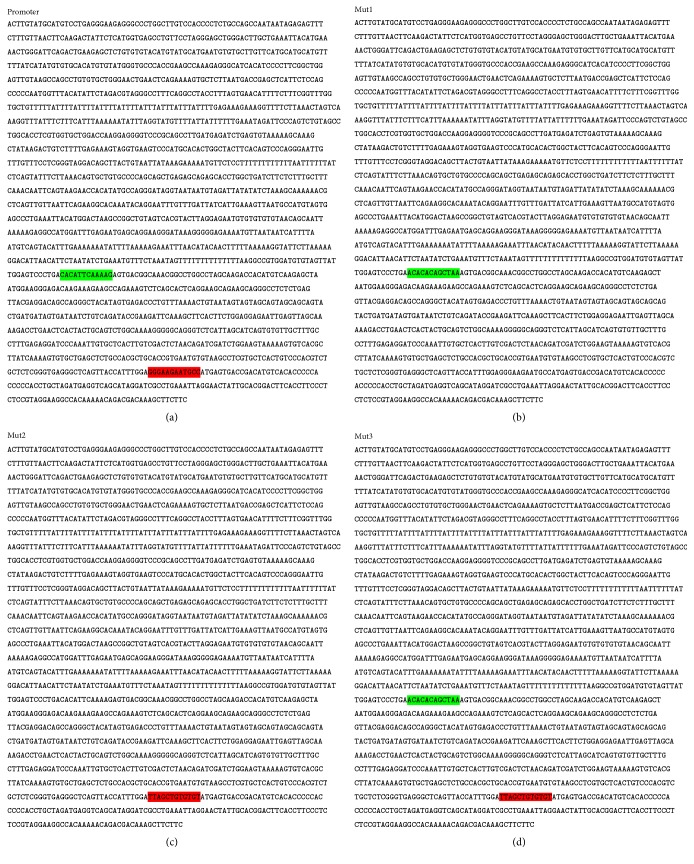
The information of promoter and mutants. (a) The promoter sequence of miR-29a is here. The green region is the first binding site and the red is the second. (b) There is the information of mut1 which alters site 1 and retains site 2. The green region shows the change. (c) There is the information of mut2 which alters site 2 and retains site 1. The red region shows the change. (d) There is the information of mut3 which alters site 1 and site 2 together. The green and red regions show the change.

**Figure 7:**
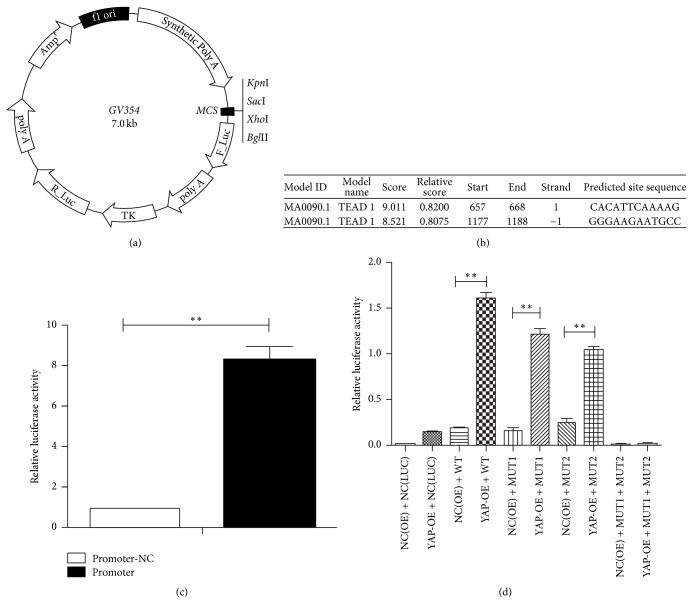
The results of luciferase report gene. (a) There are the structures of the plasmid including the promoter and the mutants. The clone sites are KpnI/XhoI. (b) The JASPAR is used to predict the YAP/TEAD binding sites and the particular contents are here. (c) There is relative luciferase activity of promoter and promoter-NC. Student's *t*-test is used here. ^*∗∗*^*P* < 0.01. (d) The relative luciferase activity of each group is here. The luciferase reporter gene analysis shows that YAP does combine directly the promoter of the miR-29a, and the binding site 2 is the major transcriptional factor binding site. An ANOVA test is used here. ^*∗∗*^*P* < 0.01.

While our data clearly establish an important role for miR-29a in axons growth, the underlying molecular mechanisms which cause the miR-29a overexpression have not been elucidated. In a previous study, we know that the YAP binds the promoter of miR-29c and the binding sites are TEAD consensus sites. Past study shows that TEAD transcription factor is the main transcription factor mediating the function of YAP. Sequence analysis of the promoter region of miR-29a reveals that the binding sites for TEAD are also contained in the promoter of miR-29a. Then, we prepare a plasmid which contains the promoter of miR-29a to test its transcriptional activity. The promoter sequence of the miR-29a in the plasmid which is searched in NCBI is showed in Figure 6(a) and the detailed information of the plasmid is presented in Figure 7(a). Subsequently, some gene sequence analyzing software is used to look for the binding sites. The JASPAR is used to predict the YAP/TEAD binding sites and the particular contents are in Figure 7(b). The information is showed in Figure 6(a) (labelled green and red). Blank plasmid vector is used as the negative control. 48 hours after the transfection, we detected the relative luciferase activity. Compared with the control group, there is 8-fold increase in luciferase relative expression (*P* < 0.05) (Figure 7(c)). The results demonstrate that the promoter of miR-29a has a strong transcriptional activity.

**Figure 8:**
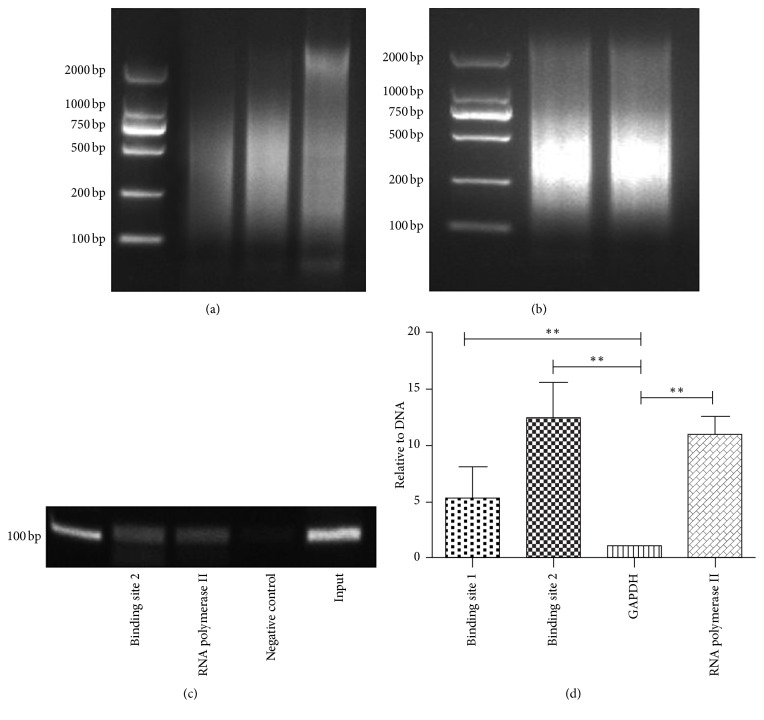
The results of a ChIP assay. (a) It is the 2% agarose gel electrophoresis with the different sonication conditions. It is 25% power, working 2 seconds, 2.5 seconds, and 1.5 seconds in sequence and pausing 8 seconds, and total time is 3 minutes. The leftmost is the marker which contains 2000 bp, 1000 bp, 750 bp, 500 bp, 200 bp, and 100 bp from top to bottom. (b) The picture exhibits the most appropriate method which is working 2.5 seconds and pausing 8 second and the total time is 3 minutes. The chromosome segment is mainly focused on from 200 bp to 500 bp. (c) The results of agarose gel electrophoresis of the samples are showed which is performed with semiquantitative PCR. From left to right, there are the marker (100 bp), the YAP antibody group (binding site 2), the RNA polymerase II antibody group, the normal IgG group, and the input group. (d) The chart reveals the quantification PCR of the samples. The result reveals that YAP binds the promoter of the miR-29a and the major binding site is the binding site 2. Mean ± SD (*n* = 3). An ANOVA test is used here. ^*∗∗*^*P* < 0.01.

After that, we prepare three mutants corresponding to the two binding sites, respectively (Figures 6(b), 6(c), and 6(d)). The original promoter of miR-29a served as a negative control. A luciferase reporter gene analysis shows the difference of luciferase relative expression (Figure 7(d)). There is a significantly decreased luciferase activity when we alter one of the two binding sites. Moreover, when we alter two binding sites at the same time, the luciferase activity almost disappeared. Subsequently, a ChIP assay is also performed to verity that YAP combines the promoter of the miR29a. After cells are treated with YAP, we collect the cells precipitate and sonicate it. The agarose gel electrophoresis is emerged with the different sonication conditions and the results are showed in Figure 8(a). The best sonication condition is 25% power, working 2.5 seconds, and pausing 8 seconds, total time is 3 minutes, and the outcome is demonstrated in Figure 8(b). Then we use the YAP antibody, anti-RNA polymerase II antibody, and normal mouse IgG to precipitate the antibody/protein/chromatin complex. At last, purified DNA is used to semiquantitative PCR and real-time PCR. The results are showed in Figures 8(c) and 8(d). The results are consistent with the bioinformatics prediction indicating that the TEAD consensus sites are the targets for YAP.

## 4. Discussion

In our study, the function of YAP on neurite outgrowth is demonstrated in N2a cells. The results provide the first evidence that YAP plays an important role in axons outgrowth via upregulating the miR-29a. Meanwhile, we have demonstrated that YAP binds the promoter of miR-29a to increase its expression.

Genetic studies have established that the Hippo-YAP pathway plays a crucial role in modulating cell proliferation and apoptosis [25]. YAP as a key downstream effector of the mammalian Hippo pathway is studied in cancer and embryonic stem cells all the time [5, 26]. However, we find that it upregulates the miR-29a by binding the promoter [21]. Since miR-29a is a newfound mediator leading to the neurite outgrowth in neurons, we cannot help but thinking of the relationship between YAP and neurite outgrowth [24]. In this study, we reveal that YAP promotes neurite outgrowth of the N2a cells. To determine whether the effect of YAP on neurite outgrowth is determined by the miR-29a, we make two steps. First, in view of the role of PTEN in axon regeneration and neuronal differentiation [12, 16] and the relationship between miR-29a and PTEN [11], a protein and gene analysis is used to test the expression of PTEN. A negative correlation is observed between YAP and PTEN. Then, we regulate the expression of miR-29a and YAP at the same time, downregulating miR-29a in YAP OE cells and upregulating miR-29a when the YAP is inhibited. After measuring the expression of PTEN and p-Akt, the downstream gene of miR-29a, we find that downregulating miR-29a can reverse the promoted axons growth of N2a cells mediated by YAP. No matter the change of YAP, the morphology change of N2a cells is dependent on the miR-29a. On the other hand, the blockage of the PI3K/Akt signaling pathway inhibits the ability that YAP promotes the axons growth. These results have revealed that the miR-29a plays a crucial role in modulating neurite outgrowth by suppressing PTEN.

Previous studies show that YAP regulates neural progenitor cell number via the TEAD transcription factor [27]. YAP itself has no DNA-binding activity; it stimulates gene expression by binding to DNA-binding transcription factors [28]. Moreover, YAP binds directly to promoters of a lot of genes known to stimulate their expression, such as miR-29c. Since the sequences of seed region of miR-29s are highly conserved, we boldly conjecture that YAP increases miR-29a by binding the promoter region of miR-29a and the key binding site is the TEAD consensus site. Some software is applied for predicting the binding site; we get two binding sites in the promoter of miR-29a. Further, we prove the strong transcriptional activity of the promoter of miR-29a by luciferase reporter gene. These three mutants also indicate that the TEAD binging sites do play a significant role in promoting expression of miR-29a. At last, we perform a ChIP assay to validate the results. The semiquantitative PCR and the real-time PCR also support the conclusion. We now provide evidences that miR-29a is an important target of YAP and YAP promotes the expression of miR-29a by binding TEAD consensus sites. Also, we have some evidences that YAP downregulates PTEN by inducing miR-29a to inhibit PTEN translation. These data indicate the way that YAP promotes the neurite outgrowth.

It should be noted that this study has examined only inN2a cells. Yet we do not know whether the promoting functions exist in other organs or animals. And this will be the key point in our next work.

## 5. Conclusion

The current work shows that YAP can increase the expression of miR-29a which can decrease the level of PTEN, a major inhibitor to suppress the axonal growth. Our work reveals that the miR-29a promotes the Akt phosphorylation to increase the neurite outgrowth. It is also noted that YAP combines directly the promoter of miR-29a. This study does reveal the ability of YAP in neurite growth and provide a promising research hint for the cure of neural disease in the future [1].

## Figures and Tables

**Table 1:** The list of primers for real-time PCR.

Primer	Sequence (5′-3′)	Base
GAPDH (forward)	GAAGGGTGGAGCCAAAAG	18
GAPDH (reverse)	ACCAGTGGATGCAGGGAT	18
PTEN (forward)	TGAAGACCATAACCCACC	18
PTEN (reverse)	CATTACACCAGTCCGTCC	18
GAP-43 (forward)	AAACAAGCCGATGTGCCT	18
GAP-43 (reverse)	CGTCTACAGCGTCTTTCTCC	20
NF-200 (forward)	GCAGACATTGCCTCCTACC	19
NF-200 (reverse)	GACACTCTTCGCCTTCCAG	19
miR-29a (forward)	GCGCACTGATTTCTTTTGGTGTTCAG	26
miR-29a (reverse)	GCGAGCACAGAATTAATACGAC	22
U6 (forward)	CTCGCTTCGGCAGCACA	17
U6 (reverse)	GCGAGCACAGAATTAATACGAC	22
miR-29a promoter (forward)	GTTATTGGAGTCCCTGAC	18
miR-29a promoter (reverse)	CTGGCTTCTTTCTTGTCT	18
Another miR-29a promoter (forward)	AAGCCTCGTGCTCACTGTCCC	21
Another miR-29a promoter (reverse)	CACTCATGGCATTCTTCCCTCC	22
